# Analysis of Ethanol Dehydration using Membrane Separation Processes

**DOI:** 10.1515/biol-2020-0013

**Published:** 2020-03-24

**Authors:** Carolina Conde-Mejía, Arturo Jiménez-Gutiérrez

**Affiliations:** 1 Tecnológico Nacional de México/Instituto Tecnológico de Celaya, Departamento de Ingeniería Química, Celaya, Gto. 38010 México; 2 Universidad Juárez Autónoma de Tabasco, División Académica Multidiciplinaria de Jalpa de Méndez, Ingeniería en Petroquímica. Carretera Estatal Libre Villahermosa- Comalcalco Km. 27+000 s/n Ranchería Ribera Alta, C.P. 86205, Jalpa de Méndez, Tabasco, México

**Keywords:** Bioethanol, Membrane separation, Ethanol dehydration, Pervaporation, Vapor permeation

## Abstract

After the biomass pretreatment and fermentation processes, the purification step constitutes a major task in bioethanol production processes. The use of membranes provides an interesting choice to achieve high-purity bioethanol. Membrane separation processes are generally characterized by low energy requirements, but a high capital investment. Some major design aspects for membrane processes and their application to the ethanol dehydration problem are addressed in this work. The analysis includes pervaporation and vapor permeation methods, and considers using two types of membranes, A-type zeolite and amorphous silica membrane. The results identify the best combination of membrane separation method and type of membrane needed for bioethanol purification.

## 1 Introduction

Because of the high purity required for bioethanol for several applications, including its use as fuel, the dehydration step represents a major challenge in its production process. One problem with this purification step is the azeotrope for the ethanol-water mixture that requires a special separation process. One option worth of consideration is the use of membrane separation arrangements to carry out such ethanol purification process, for which two methods are typically considered, namely pervaporation and vapor permeation. Several characteristics of membrane separation processes are first described, followed by a review on reported works for ethanol dehydration using membranes.

**Figure 1:**
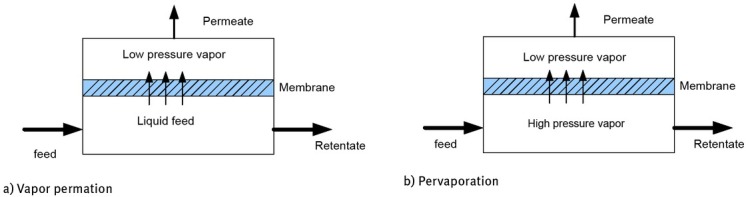
Pervaporation and vapor permeation representation

The difference between the pervaporation (PV) and vapor permeation (VP) separation processes relies on the feed condition, liquid for PV and vapor for VP. The feed stream side is at high pressure, while the other side is at low pressure, producing a low-pressure vapor. The vapor generated is called permeate, while the stream that remains in the feed side is known as retentate. Figure 1 shows the two processes. Although the mass transport phenomena through the membrane are not totally well known, the solution-diffusion model is frequently adopted, because it has been shown to provide good approximations for the behavior of membrane separation systems [1, 2]. Some variations of the model have also been developed [3, 4, 5, 6].

Important applications of PV and VP for solvent dehydration using hydrophilic membranes have been reported [7, 8, 9, 10]. Also, the organic mixture separation has started to be reported as an application area of these methods [11, 12]. The success of their industrial implementation strongly depends on the membrane materials. Although the membrane separation started at the end of 1960 with reverse osmosis, it was not until 1980 when the first industrial application for gas separation mixture was implemented, due to the start of serial production of commercial polymeric membranes [13, 14]. Polymeric membranes have been successfully applied; however, its intrinsic limitations, such as low temperature and mechanic resistance excluded its use for several applications. More recently, inorganic membranes have been used; they can operate at higher temperatures and show better mechanic resistance than polymeric membranes, but they are generally more expensive [15, 16].

Membrane separations are used for azeotropic or very close boiling point mixtures when conventional processes, such as distillation, adsorption or absorption, need high energy consumption or complex configurations. However, in order to avoid high area requirements it is convenient to combine membrane separation methods with conventional separation processes; several works have addressed this approach through the integration of distillation and membrane separation systems [17, 18, 19, 20, 21]. Design methods applied as part of optimization models for hybrid distillation-pervaporation and distillation-vapor permeation systems have also been reported [22, 23, 24, 25]. Other configurations have been recently considered, such as the use of nano-filtration membranes [26], the integration of solar-driven membranes with distillation [27], and the novel development of membrane bioreactors [28, 29, 30].

One of the limitations of ethanol fuel production from removable sources is the high purity required because of the need for a costly separation process [31, 32, 33]. Distillation followed by a membrane module can be considered to reduce the ethanol purification cost [22,33, 34, 35, 36]. The membrane module can operate in pervaporation or vapor permeation fashion. Several questions have to be answered in order to implement these hybrid processes, including the selection of the best option (PV or VP), operating conditions, and type of membrane to be used. In this work, we address these items through a systematic methodology. Our analysis involved the PV and VP processes and two types of inorganic and hydrophilic membranes, the A-type zeolite and amorphous silica.

## 2 Methodology

**Figure 2:**
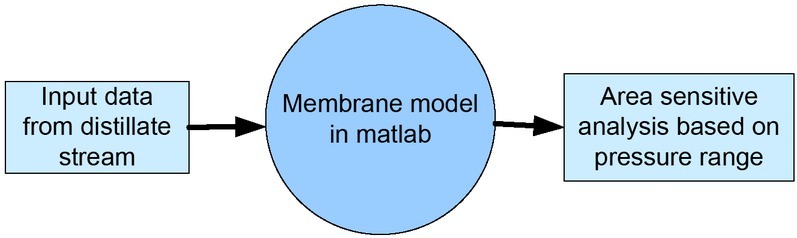
General methodology

A membrane model (MM) was developed and implemented into the Matlab environment to calculate the area requirements for PV and VP systems. The MMs involved the solution of the mass and energy balance for the membrane. We assumed that the feed stream to the membrane is the distillate from a binary column; then, the simulation of ethanol-water mixture distillation was implemented in Aspen plus in order to get the distillate conditions, which become the MM input data. A membrane area sensitive analysis was developed by varying the feed pressure and the permeate pressure on the membrane. A general methodology representation is shown in Figure 2.

### 2.1 Input data from distillation simulations

**Figure 3:**
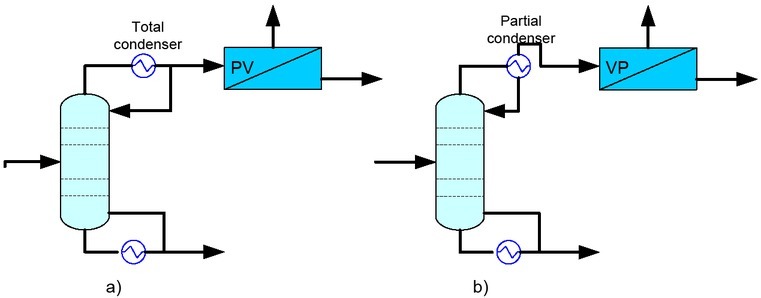
Condenser options, a) total condenser before PV; b) partial condenser before VP

A conventional distillation column was implemented into Aspen plus, using RadFrac subroutine and the NRTL thermodynamic model. The column feed was defined as a saturated liquid of an ethanol-water mixture with a molar flow of 140.45 Kmol/hr and 0.3316 ethanol mol composition (> 50 wt%). For the distillate stream the ethanol recovery and purity were input as design specifications with 99.09% and 92.01% (81.81% mol) purity, based on initial ethanol mass flow. These conditions are below the azeotropic composition. The mass reflux ratio and mass distillate rate were set as process parameters. The distillation column was set with 15 equilibrium stages, with feed in tray 12. A total condenser was used to get the PV input data and a partial condenser was used to get the VP input data. A schematic representation is shown in Figure 3.

In order to get the distillate conditions at different pressures, a set of pressure condensers was defined. Table 1 shows the pressure and the distillate temperatures for the PV and VP processes; for the PV process the liquid enthalpy is required. The distillate molar flow was 56.5 Kmol/hr with 0.8181 ethanol mol composition, for all cases.

**Table 1:** Results for distillate stream (membrane input data)

PF (bar)	T for PV (K)	h^L^ for PV (KJ/Kmol)	T for VP (K)
1.52	361.6	-270572	362.0
1.72	365.1	-270083	365.4
2.03	369.7	-269431	370.0
2.33	373.7	-268838	374.0
2.96	380.9	-267771	381.2
3.45	385.7	-267045	385.9
4.05	390.8	-266245	391.1

### 2.2 Membrane model

**Figure 4:**
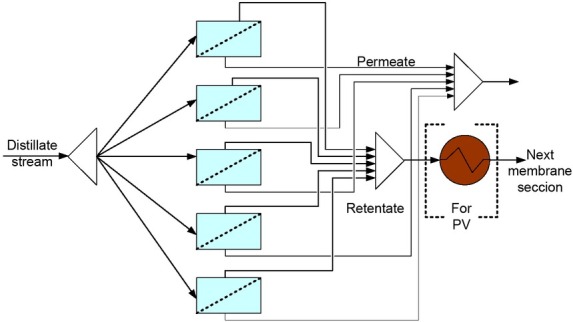
Membrane section arrangement

The membrane separation section shown in Figure 4 was considered. For the VP method only one separation section was required, with five membrane modules and a maximum area for each module of 500 m^2^. In the case of the PV method more than one separation section could be required; in this case, a heat exchanger was needed between the two separation sections. Each section had five membrane modules with a maximum area of 100 m^2^, and up to 10 sections.

The total distillate flow was divided into five equal sub-streams, one for each module, with an individual flow of 11.3 Kmol/hr.

#### 2.2.1 Modeling equations

Pervaporation and vapor permeation processes can be described by the solution-diffusion model [1, 2], but they have different driving forces. In the VP case, since the two side of membrane are in the vapor phase, the driving force was defined as a partial pressure difference. For PV processes, Wijmans and Baker [2] proposed the driving force as a vapor pressure difference. Therefore, the equation (1) defines the molar flux, *J_i_*, through the membrane for the VP method and equation (2) gives the molar flux for the PV process.

(1) $$J_{i}=Q_{i}{}^{⋆}\left(x_{i,o}P_{o}-y_{i,l}P_{p}\right).$$

(2) $$J_{i}=Q_{i}*\left(x_{i,0}\gamma_{i,0}p_{i}{,}_{0}^{sat}-y_{i,l}{\text{P}}_{p}\right).$$

The permeance (*Q_i_*) is a property for each component in one kind of membrane, which relates the permeability coefficient and the membrane thickness. According to Sommer and Melin [10], for A-type zeolite and amorphous silica membrane the permeance can be estimated as a temperature function using the following relation,

(3) $$Q_{i}=Q_{i,ref}exp\left[\frac{E_{i}}{R}\left(\frac{1}{T_{ref}}-\frac{1}{T_{F}}\right)\right]$$

where the reference temperature (*T_ref_*) is 80°C, and the parameters *Q_i,ref_* and *E_i_* have to be known for each component. The values for the ethanol-water system that were reported by Sommer and Melin [10] were used in this work.

In order to find the permeate and the retentate flows, output compositions, and in the PV case the temperature drop through the membrane, a differential equation model was used, with variables expressed as a function of the membrane area. The model is based on the shortcut method proposed by Bausa and Marquardt [19].

In the VP process, no latent heat is required in the transport of the components because there is not a phase change; then, the temperature drop through the module was neglected [18]. For the VP case, only a mass balance is required. Equation (4) gives the vapor flow change in the retentate, equation (5) the mol fraction change for each component for the retentate side, while equation (6) relates the permeate composition with the total flux and component flux.

(4) $$\frac{dV}{dA}=-J_{T}$$

(5) $$\frac{dx_{i}}{dA}=-\frac{J_{T}}{V}\left(y_{p,i}-x_{i}\right)$$

and

(6) $$y_{p.i}=\frac{J_{i}}{J_{T}}$$

The boundary conditions are:

(7) $$V\left(A=0\right)=F$$

(8) $$x_{i}\left(A=0\right)=x_{i,F}$$

(9) $$V\left(A_{t}\right)=R$$

(10) $$x_{i}\left(A_{t}\right)=x_{R}$$

In the PV process there is a phase change through the membrane; the energy required comes from the liquid in the retentate side. An enthalpy change in the liquid phase gives a temperature drop through the membrane module. The temperature drop reduces the mass transfer through the membrane; therefore, it is required to re-heat the retentate stream between two modules in order to achieve the desired separation. Mass and energy balance must be solved simultaneously. The following equations represent the PV model. Equations (11) and (12) give the liquid flow and component composition change for the retentate stream, while equation (13) gives the enthalpy change for the liquid.

(11) $$\frac{dL}{dA}=-J_{T}$$

(12) $$\frac{dx_{i}}{dA}=-\frac{J_{T}}{L}\left(y_{p,i}-x_{i}\right)$$

(13) $$\frac{dh^{L}}{dA}=-\frac{J_{T}}{L}\left(h^{v}-h^{L}\right)$$

The boundary conditions are:

(14) L(A=0)=F

(15) $$x_{i}\left(A=0\right)=x_{i,F}$$

(16) $$h^{L}\left(A=0\right)=h_{F}^{L}$$

(17) $$L\left(A_{t}\right)=R$$

(18) $$x_{i}\left(A_{t}\right)=x_{R}$$

(19) $$h^{L}\left(A_{t}\right)=h_{R}^{L}$$

In order to solve equation (13), liquid and vapor enthalpies for ideal mixtures were assumed [37].

(20) $$h^{L}=\sum_{i=1}^{c}x_{i}\left(h_{i,v}^{0}-\Delta H_{i}^{vap}\right)$$

(21) $$h^{v}=\sum_{i=1}^{c}y_{i}h_{i,v}^{0}$$

Using equations (20) and (21), one can write the energy balance with Equation (13) for a binary system as,

(22) $$\frac{dh^{L}}{dA}=-\frac{J_{T}}{L}\left[h_{1,v}^{0}\left(y_{1}-x_{1}\right)+h_{2,v}^{0}\left(y_{2}-x_{2}\right)+x_{1}\Delta H_{1}^{vap}+x_{2}\Delta H_{2}^{vap}\right]$$

The PV model assumes a saturate vapor phase on feed side; then, the equilibrium condition between the liquid phase and the saturate vapor is given by *y_i_*=*k_i_x_i_*. On the order hand, the composition is close to the azeotropic condition; therefore, *k_i_* values are close 1.0 and it can be assumed that *y_i_‒x_i_*≈0. Applying this condition to equation (22), one obtains,

(23) $$\frac{dh^{L}}{dA}=-\frac{J_{T}}{L}\left[x_{1}\Delta H_{1}^{vap}+x_{2}\Delta H_{2}^{vap}\right]$$

Equation (23) was used to solve the liquid enthalpy change for the PV process.

#### 2.2.2 Matlab structure

**Figure 5:**
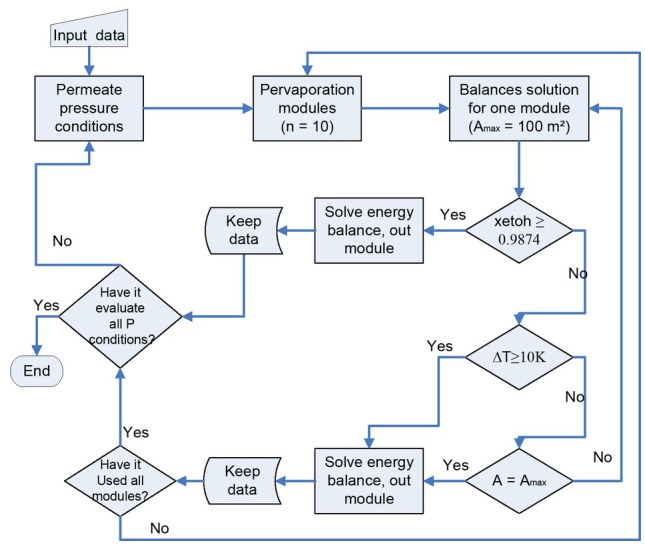
Matlab algorithm to solve the PV system

**Figure 6:**
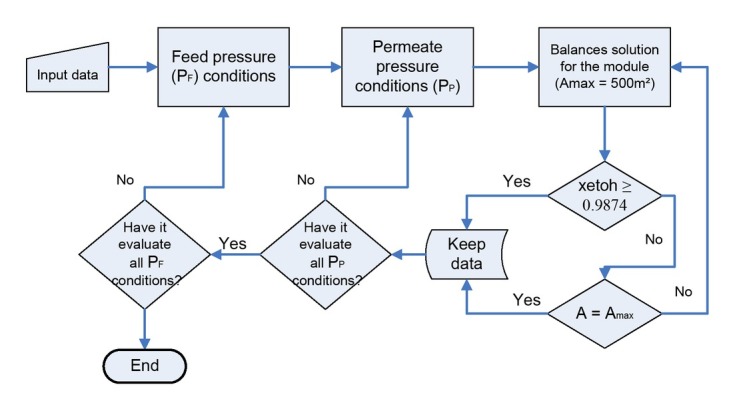
Matlab algorithm to solve the VP system

The membrane model was solved aided by Matlab for each the PV and VP cases. Figures 5 and 6 show the algorithm implemented in order to solve the balances for the membrane modules.

In the MM for PV (Figure 5) three cycles were used, the first one solves the balances for one module, the second one solves the balances for several modules and the last one gets the solutions for a set of permeate pressure conditions. The feed pressure conditions were manually changed for each run. Three physical restrictions were used to stop the first cycle. First, if the ethanol mol composition is equal or higher than 0.9874, the total area required has been found and a new run is started for a new permeate pressure condition. Second, a maximum drop temperature for one module was defined as 10 K; if the temperature drop is equal or higher than this value, the balances are solved in a new module. Finally, the maximum area for one module was defined as 100 m^2^; when this value is reached in one module without achieving the target composition, a new module is needed to continue the solution process.

The logic used in VP MM (Figure 6) was similar to PV MM. Three cycles were also used, the first one solves the balances for one module, the second one gets the solution for a set of permeate pressures and the last one gets the solution for a set of feed pressures. In the VP case, only one membrane section was used, with not re-heat required for the retentate stream. Table 2 shows the input and output data for the MM for each system.

**Table 2:** Input and output data for MM in Matlab

Input data	Output data
Total molar flow	Permeate molar flow
Ethanol mole fraction	Permeate compositions
Water mole fraction	Retentate molar flow
Feed pressure	Retentate compositions
Feed temperature	Permeate temperature
Liquid enthalpy for PV case	Retentate temperature

## 3 Results and Discussion

### 3.1 A-type zeolite results

Table 3 shows the membrane area required and number of modules for the PV method. The first table column shows the permeate pressure and the first table row shows the feed pressure (retentate pressure). For the PV case, when the number of modules was higher than 10, areas were not reported. Table 4 shows the area required for the VP method, using the A-type zeolite membrane. In the VP case, when the area required for one module was higher than 500 m^2^ (higher than 2500 for section), areas were not reported.

**Table 3:** Area (m^2^) and modules (area/ modules) required for A-type zeolite membrane using the PV method

P_P_/P_0_ [bars]	4.05	3.45	3.04	2.33	2.03	1.72	1.52
0.05	105/ 4	325/ 5	295/ 5	945/ 6	1485/ 7	2760/ 9	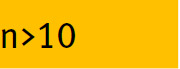
0.06	190/ 4	555/ 5	560/ 5	1540/ 7	2420/ 9	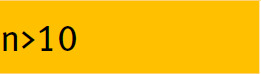	
0.07	360/ 4	670/ 5	1035/ 6	2310/ 8	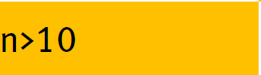		
0.08	570/ 5	890/ 5	1440/ 6	3270/ 10			
0.09	705/ 5	1170/ 6	1920/ 7	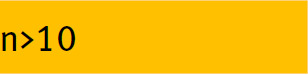			
0.1	890/ 5	1495/ 6	2480/ 8				
0.11	1115/ 6	1855/ 7	3010/ 9				
0.12	1255/ 6	2245/ 8	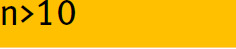				
0.13	1520/ 6	2700/ 9					
0.14	1815/ 7	3190/ 10					

**Table 4:** Area (m^2^) required for A-type zeolite membrane using the VP method

P_P_/P_0_ [bars]	4.05	3.45	3.04	2.33	2.03	1.72	1.52
0.05	95	160	305	875	1555	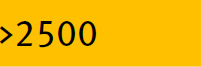	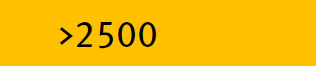
0.06	140	285	565	1545	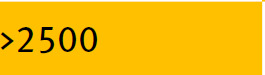		
0.07	235	485	935	2405			
0.08	375	750	1400	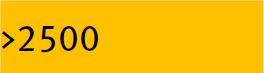			
0.09	555	1080	1950				
0.1	775	1465	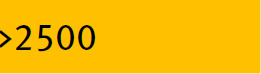				
0.11	1035	1900					
0.12	1325	2385					
0.13	1645	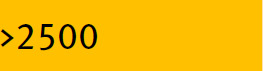					
0.14	1995						

According to these results, high pressure differences are needed in order to achieve low areas values. In general, the VP method needs lower areas than the PV method; moreover, re-heating the retentate stream for the VP method is not needed.

### 3.2 Amorphous silica membrane results

Table 5 gives the areas and number of modules for the PV method, while Table 6 shows the results for the VP case using the amorphous silica membrane. For this kind of membrane, lower areas are also required with the VP method. Lower areas are obtained using the amorphous silica membrane than using the A-type zeolite membrane.

**Table 5:** Area (m^2^) and modules (area/ modules) required for amorphous silica membrane using the PV method

P_P_/P_0_ [bars]	4.05	3.45	3.04	2.33	2.03	1.72	1.52
0.05	65/ 4	85/ 5	105/ 5	180/ 5	240/ 6	325/ 6	440/ 6
0.06	75/ 5	95/ 5	120/ 5	215/ 6	285/ 6	410/ 6	575/ 7
0.07	75/ 5	100/ 5	140/ 5	240/ 6	330/ 6	505/ 7	680/ 7
0.08	85/ 5	110/ 5	155/ 5	285/ 6	390/ 6	605/ 7	830/ 7
0.09	90/ 5	125/ 5	185/ 5	320/ 6	480/ 6	700/ 7	990/ 8
0.1	95/ 5	140/ 5	215/ 5	370/ 6	530/ 7	835/ 7	1140/ 8
0.11	115/ 5	155/ 5	240/ 6	430/ 6	605/ 7	950/ 8	1360/ 8
0.12	120/ 5	180/ 5	260/ 6	495/ 7	695/ 7	1080/ 8	1515/ 9
0.13	130/ 5	205/ 5	285/ 6	540/ 7	810/ 8	1245/ 9	1750/ 9
0.14	140/ 5	220/ 5	315/ 6	600/ 7	880/ 8	1375/ 9	1930/ 10

**Table 6:** Area (m^2^) required for amorphous silica membrane using the VP method

P_P_/P_0_ [bars]	4.05	3.45	3.04	2.33	2.03	1.72	1.52
0.05	60	75	95	145	190	260	335
0.06	65	85	110	170	220	315	410
0.07	70	90	120	195	260	375	495
0.08	75	100	135	225	305	440	590
0.09	85	115	155	255	350	515	690
0.1	90	125	170	290	400	595	805
0.11	100	135	190	330	455	680	920
0.12	105	150	210	370	515	770	1045
0.13	115	165	235	415	575	865	1180
0.14	125	180	260	455	640	965	1315

### 3.3 Comparison between A-type zeolite membrane and amorphous silica membrane

**Figure 7:**
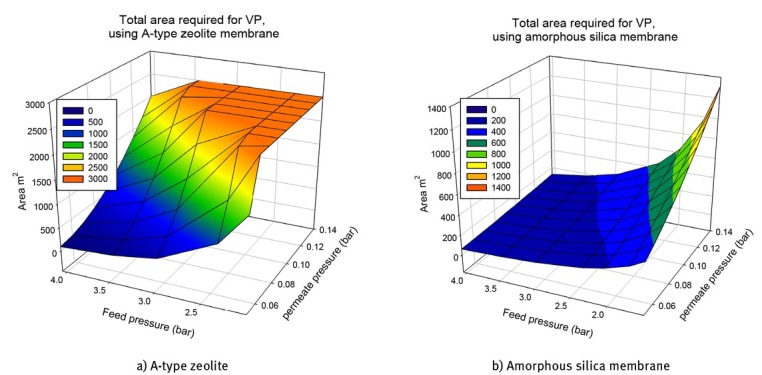
Membrane area required as a function of pressure differential

In this section, the VP results for the two types of membranes were plotted in order to compare the membrane performances. Figure 7 shows the two membrane plots (the flat zone in Figure 7 corresponds to the areas not reported). It can be observed that A-type zeolite membrane has a higher sensitivity to delta pressure decrease than amorphous silica membrane. This means that amorphous silica membrane can operate with lower feed pressure and higher permeate pressure, which provides lower operation costs.

**Figure 8:**
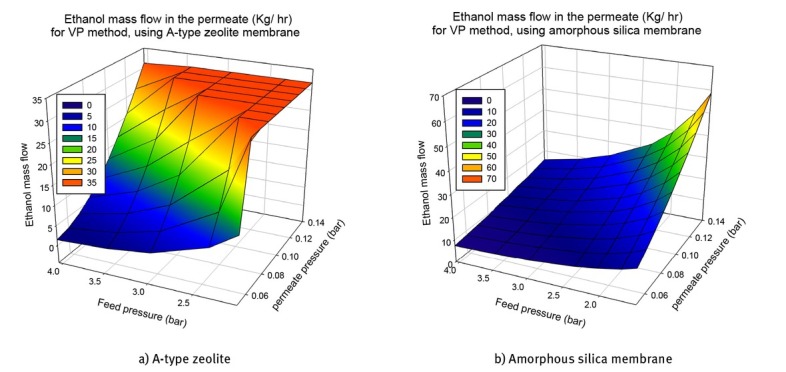
Ethanol mass flow in permeate stream

Figure 8 shows the ethanol mass flow leaving with the permeate stream for the two types of membranes using the VP method. It can be observed that the mass flow performance is similar to the area performance; however, for higher pressure differences the A-type zeolite gives lower ethanol mass flows in the permeate. These results agree with the fact that the A-type zeolite is a more selective membrane than the amorphous silica membrane. Moreover, it can be inferred that silica membranes will need to operate with a permeate recycle to the distillation column, while for A-type zeolite membranes use the recycle implementation could be not necessary.

**Figure 9:**
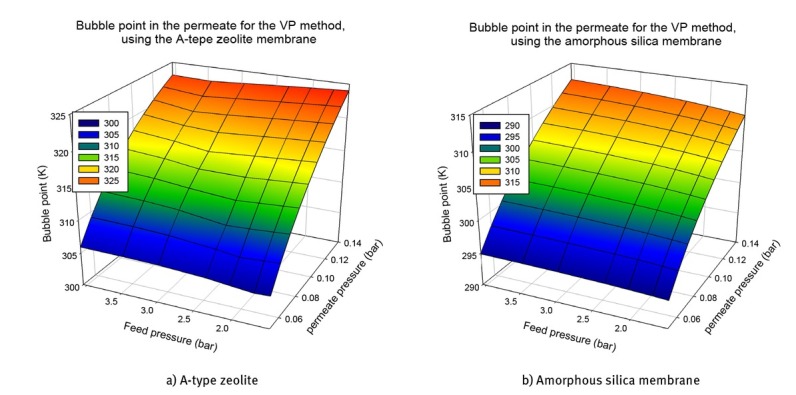
Results for the VP method using the two membranes

Finally, in order to select the permeate pressure, the bubble point temperature for the permeate stream should be taken into account. Since the low-pressure vapor stream in the permeate needs to be condensed, it will be convenient to use cooling water instead of a refrigerant. Based on the permeate composition, we estimated the bubble point temperature. The results are reported in Figure 9 for the VP method using the two membranes. The bubble point using the PV method was also estimated, and the performance was very similar to the use of VP. It can be observed that the bubble point temperature only depends on the permeate pressure. Based on these results, permeate pressures higher than 0.08 bar for A-type zeolites and higher than 0.13 bar for amorphous silica membranes are recommended. Table 7 resumes the best operation conditions for each case. The lower area values were chosen with the restriction of using cooling water for the permeate condensation.

**Table 7:** Best operation conditions selected for each case

Variable	A-type zeolite membrane		Amorphous silica membrane	

	PV	VP	PV	VP
P_P_ (bar)	0.08	0.08	0.13	0.13
P_0_ (bar)	4.05	4.05	4.05	4.05
A_T_ (m^2^)	570	375	130	115
P_EtOH_ (Kg/hr)	7.29	5.91	70.73	71.31
T_b_P_ (K)	314	314	311	311
# Modules	5		5	
Permeate recycle			necessary	necessary

The results of this work show that if membrane processes are considered, the use of the VP method using an amorphous silica membrane with a recycle of the permeate stream provides an excellent option for ethanol purification.

## 4 Conclusions

The use of membrane separation systems for ethanol dehydration has been presented. A systematic methodology was developed in order to determine the feed and permeate pressure influence on the PV and VP methods for the production of high-purity ethanol. A-type zeolites and amorphous silica membranes were considered. Relevant factors such as total area requirements and membrane output conditions were determined with this procedure. As a result, the best operating conditions for the membrane separation process were identified. Based on the results, the use of VP method with amorphous silica membrane is recommended for the dehydration of bioethanol.

## Nomenclature

*J_i_*: Molar flux through the membrane, Kmol/ m^2^ h
*Q_i_*: Permeace coefficient, Kmol/ m^2^ h bar
*x_i,0_*: Mole fraction in feed side
*y_i,l_*: Mole fraction in permeate side
*P_0_*: Feed pressure, bar
*P_P_*: Permeate pressure, bar
$p_{i,0}^{sat}$: Saturation pressure at liquid conditions, bar
*γ_i,0_*: Activity coefficient
